# External stenting of saphenous vein bypass grafts does not affect intraoperative transit-time flow measurement

**DOI:** 10.1186/1749-8090-10-S1-A297

**Published:** 2015-12-16

**Authors:** Burak Can Depboylu, Patrick O Myers, Mootoosamy Parmeseeven, Jalal Jolou, Kamran Ahmadov, Saziye Karaca, Marck Licker, Afksendiyos Kalangos, Mustafa Cikirikcioglu

**Affiliations:** 1 Division of Cardiovascular Surgery, University Hospitals and Medical Faculty of Geneva, Geneva, Geneva, 1211, Switzerland; 2 Division of Anaesthesiology, University Hospitals and Medical Faculty of Geneva, Geneva, Geneva, 1211, Switzerland

## Background/Introduction

Saphenous vein grafts (SVG) are the most commonly used conduits for coronary artery bypass operations (CABG), despite their sub-optimal long-term patency. External stenting of SVG (eSVS^® ^mesh) was recently proposed to improve their long term patency. Transit time flow measurement (TTFM) is a well described method for intraoperative quality control for CABG.

## Aims/Objectives

The aim of this study is to assess whether external stenting of SVG affects perioperative TTFM.

## Methods

Twenty six patients who underwent elective CABG were divided into two groups based usage of externally stented SVG (eSVS^® ^mesh, n = 13), or bare SVG (n = 13). The anastomotic quality were evaluated with TTFM using the Medi-Stim VeriQ flowmeter and a 4 mm probe. Perioperative data were given as median (min - max) and compared between groups (Table 1).

**Table 1:** 

	Mesh covered SVG (n = 13)		Bare SVG (n = 13)		P
		
	median	min-max	median	min-max	
Age (years)	64	51-82	64	59-80	>0.05*

Bypass grafts	3	1-4	3	2-4	>0.05*

Simultaneous concomitant operation	3 (23%)		2 (15%)		>0.05°

CBP time (min)	112	57-161	94	52-134	>0.05*

Cross Clamp Time (min)	69	34-122	63	28-96	>0.05*

TTFM (ml/min)	59	19-106	43	30-155	>0.05*

PI	1.9	1.2-4.9	2.3	1.3-2.9	>0.05*

*Mann-Whitney U Test, ° Fisher's exact test

## Results

There was no significant difference between two groups regarding pre and peri-operative parameters, although more patients in the eSVS^® ^mesh group had concomitant procedures (3, 23% vs. 2, 15%, P > 0.99). All SVG were patent in both groups at the end of the surgical procedure and TTFM values were similar. eSVS^® ^mesh group had a trend for longer cardiopulmonary and aortic cross clamping times, which didn't reach statistical significance.

## Conclusion

External stenting of SVG by eSVS^® ^mesh does not extend the operative times. All SVG showed excellent flow and eSVS^® ^mesh coverage didn't impede TTFM or provide graft flow different to controls.

